# Mixed housing with DBA/2 mice induces stress in C57BL/6 mice: implications for interventions based on social enrichment

**DOI:** 10.3389/fnbeh.2014.00257

**Published:** 2014-08-06

**Authors:** Natalia Kulesskaya, Nina N. Karpova, Li Ma, Li Tian, Vootele Voikar

**Affiliations:** Neuroscience Center, University of HelsinkiHelsinki, Finland

**Keywords:** animal model, stress, mixed housing, stress-related genes, behavioral interventions, social enrichment, social learning

## Abstract

Several behavioral interventions, based on social enrichment and observational learning are applied in treatment of neuropsychiatric disorders. However, the mechanism of such modulatory effect and the safety of applied methods on individuals involved in social support need further investigation. We took advantage of known differences between inbred mouse strains to reveal the effect of social enrichment on behavior and neurobiology of animals with different behavioral phenotypes. C57BL/6 and DBA/2 female mice displaying multiple differences in cognitive, social, and emotional behavior were group-housed either in same-strain or in mixed-strain conditions. Comprehensive behavioral phenotyping and analysis of expression of several plasticity- and stress-related genes were done to measure the reciprocal effects of social interaction between the strains. Contrary to our expectation, mixed housing did not change the behavior of DBA/2 mice. Nevertheless, the level of serum corticosterone and the expression of glucocorticoid receptor *Nr3c1* in the brain were increased in mixed housed DBA/2 as compared with those of separately housed DBA/2 mice. In contrast, socially active C57BL/6 animals were more sensitive to the mixed housing, displaying several signs of stress: alterations in learning, social, and anxiety-like behavior and anhedonia. These behavioral impairments were accompanied by the elevated serum corticosterone and the reduced expression of *Nr3c1*, as well as the elevated *Bdnf* levels in the cortex and hippocampus. Our results demonstrate the importance of social factors in modulation of both behavior and the underlying neurobiological mechanisms in stress response, and draw attention to the potential negative impact of social interventions for individuals involved in social support.

## Introduction

Social environment is one of the most powerful factors affecting both physiology and behavior from early childhood throughout the lifespan. Routine daily experience of social interactions slowly shapes psychological and physiological parameters of an organism and could determine its further development, reaction to stress and sensitivity to disorders. Pathways involved in regulation of the social and emotional behavior, cognitive functions, stress response, and activation of the immune system are behind the effects of social factors on physiology and psychology (Devries, 2002; Karelina et al., 2009; Karelina and Devries, 2011). Permanent social stress results in prolonged activation of the hypothalamic–pituitary–adrenal (HPA) axis and subsequently in glucocorticoid desensitization. Another potential neuroendocrine mechanism may involve oxytocin, a hypothalamic hormone that has been shown to modulate both affective behaviors and stress response (Benarroch, 2013). Besides, brain-derived neurotropic factor (BDNF) plays an important role in adult brain plasticity. Functional polymorphism in *BDNF* gene was recently shown to moderate social stress sensitivity (Perkovic et al., 2014). In spite of extensive research exploring the separate signaling pathways in mood regulation, social and cognitive behaviors, and stress response, the overall picture of neurobiological events taking place in behavioral and brain pathologies needs still further investigation.

Alterations in lifestyle and social environment could be an effective and simple way to improve behavior in pathological conditions. People suffering from dementia and loss of declarative memory can learn new motor skills for everyday household activity by observation (van Tilborg et al., 2011). Social support and collaboration with familiar healthy communicational partner improve the learning of new tasks in patients with hippocampal amnesia and Alzheimer's disease (Duff et al., 2013). Moreover, cognitive training, family support, and observational learning also improve some symptoms in patients with schizophrenia (Kern et al., 2009). Peer-mediated social engagement, observational learning and training of social skills are especially promising for treatment of abnormal social behaviors and communications in children with autism (Reichow and Volkmar, 2010; Kaplan and McCracken, 2012).

While there are a lot of investigations on the effectiveness of social intervention in ameliorating behavioral pathologies, the safety of the applied intervention for healthy subject(s) involved in social enrichment has been rarely evaluated in human and animal studies. Nevertheless, long-term interaction with affected subjects could provide a negative social experience and affect the welfare and behavior of healthy family members and caregivers (Steele et al., 2010; Sadowsky and Galvin, 2012; Wittenberg et al., 2013). It was found that healthy siblings of children with chronic disease are at risk of developing mood disorders, such as depression or anxiety and psychosis (Sharpe and Rossiter, 2002; Arajarvi et al., 2006; O'Brien et al., 2009).

Animal models as a translational tool provide good opportunities for well-controlled multifaceted studies to evaluate the mechanisms of human behavioral abnormalities and to validate different behavioral interventions or therapies. The most popular animal models to explore the role of social factors in behavior and pathology include crowding, social isolation, social instability, social defeat due to exposure to aggressive conspecific and communal nesting (Haller et al., 2004; Voikar et al., 2005; Branchi et al., 2010; Golden et al., 2011; Kulesskaya et al., 2011; Razzoli et al., 2011; Slattery et al., 2012). However, normal social interaction of animals housed together could also affect the behavior of each other and has been used as an animal model of behavioral intervention based on social enrichment. BTBR T+tf/J (BTBR) is an inbred strain validated as a model of autism-like behavior (McFarlane et al., 2008) and Yang with colleagues tried to apply peer- and parents-mediated interventions in BTBR mice. Paired housing with socially active C57BL/6J as cage-mates, but not cross-fostering with C57BL/6J, was effective in improvement of social functions of BTBR adolescent mice (Yang et al., 2007, 2011). Re-socialization (group-housing with normally developed peers) rescued the prosocial deficit induced by post-weaning social isolation in rats (Tulogdi et al., 2014). The effectiveness of group-learning and social enrichment in facilitation of learning and memory was also validated in an animal model of Alzheimer's disease (Kiryk et al., 2011) and in BTBR mice (Lipina and Roder, 2013).

Our work aimed at exploring reciprocal effects of co-housing animals with contrasting behavioral phenotypes on their behavior and neurobiological pathways. DBA/2 and C57BL/6 mice were selected since they are the most extensively studied inbred strains. They show different phenotypes in many behavioral categories—learning and memory (Upchurch and Wehner, 1988; Holmes et al., 2002), anxiety-like behavior (Holmes et al., 2002; Voikar et al., 2005), sensorimotor gating (Olivier et al., 2001; Singer et al., 2009), addiction and reward-related behavior (Tolliver and Carney, 1994; Fish et al., 2010), attention and impulsivity (Pattij et al., 2007; Pinkston and Lamb, 2011), as well as reaction to stressful manipulations (Cabib et al., 2002; Voikar et al., 2005; Mozhui et al., 2010). DBA/2 mice demonstrate deficits in spatial memory, emotional and social behavior (Bouwknecht and Paylor, 2002; Moy et al., 2008; Singer et al., 2009), in comparison with C57BL/6 mice. Therefore, we performed a comprehensive phenotyping of social, emotional, and cognitive behaviors, and gene expression analysis in female mice of these two strains, which were housed in a separated (only one strain in a cage) or mixed group (both strains together in a cage). A combination of home-cage monitoring in an IntelliCage system with the classical individual tests allowed us to explore different aspects of mouse behavior and to get an insight in the underlying modulatory processes. Based on the above mentioned works demonstrating the effectiveness of social enrichment and observational learning, we expected to find some positive effects of mixed housing on behaviors of DBA/2 mice. Furthermore, since behavior of “normal” animals involved in such social interaction has been rarely explored, we aimed to study whether mixed housing had any effect, positive or negative, on C57BL/6 mice.

## Materials and methods

### Animals

Altogether, 28 DBA/2JRccHsd (DBA/2) and 28 C57BL/6NHsd (BL6) female mice from commercial breeder (Harlan, The Netherlands) were used. The mice arrived at the age of 7 weeks and were randomly assigned to the groups of 4–5 mice to adapt for 3 weeks in standard Type III cages (with aspen chips bedding and nesting material, food and water available *ad libitum*). Animals were maintained at standard controlled conditions (room temperature 21 ± 1°C, related humidity 50–60%) on a 12 h light/dark cycle (lights on at 6 a.m.). RFID transponder (T-IS 8010 FDX-B, DATAMARS, Switzerland) was implanted subcutaneously on the nape of each mouse one week before the experiment started. The experiment was designed as two-factorial—strain (BL6, DBA/2) and housing condition (mixed, separate)—as between-subjects factor. At the age of 10 weeks the mice were rearranged into experimental groups: 2 or 3 animals from 4 original cages were jointing into one IntelliCage to form new separate (8 mice/IntelliCage) or mixed group (12 mice/IntelliCage, 6 BL6 + 6 DBA/2), respectively. For each condition (BL6 separate, DBA/2 separate, BL6+DBA/2 mixed), two IntelliCages as two independent replicates were used. Altogether, the number of animals in different subgroups was as follows: BL6-separate *n* = 16, DBA/2-separate *n* = 16, BL6-mixed *n* = 12, DBA/2-mixed *n* = 12. Immediately after regrouping, animals were placed into IntelliCages where they spent the next 45 days. Detailed description of IntelliCage (NewBehavior AG, Zurich, Switzerland) can be found elsewhere (Krackow et al., 2010; Kulesskaya et al., 2013). Briefly, four triangular operant chambers located in each corner of the cage had automatically controlled doors (2 per corner) to provide access to the nipples of drinking bottles through the round opening (13 mm diameter). The chambers provide space for one mouse at a time and every individual mouse was identified by antennas and presence sensors located in operant chambers. The IntelliCage system detected the number and duration of visits to every operant chamber, as well as the number of lickings at each bottle. During testing in the IntelliCage, the animals were handled by experimenter only after the 1st and 3rd week for cage cleaning and body weight measuring. All animal experiments were approved by the County Administrative Board of Southern Finland (ESAVI-2010-09011/Ym-23).

### Intellicage protocols

**Figure 1:**
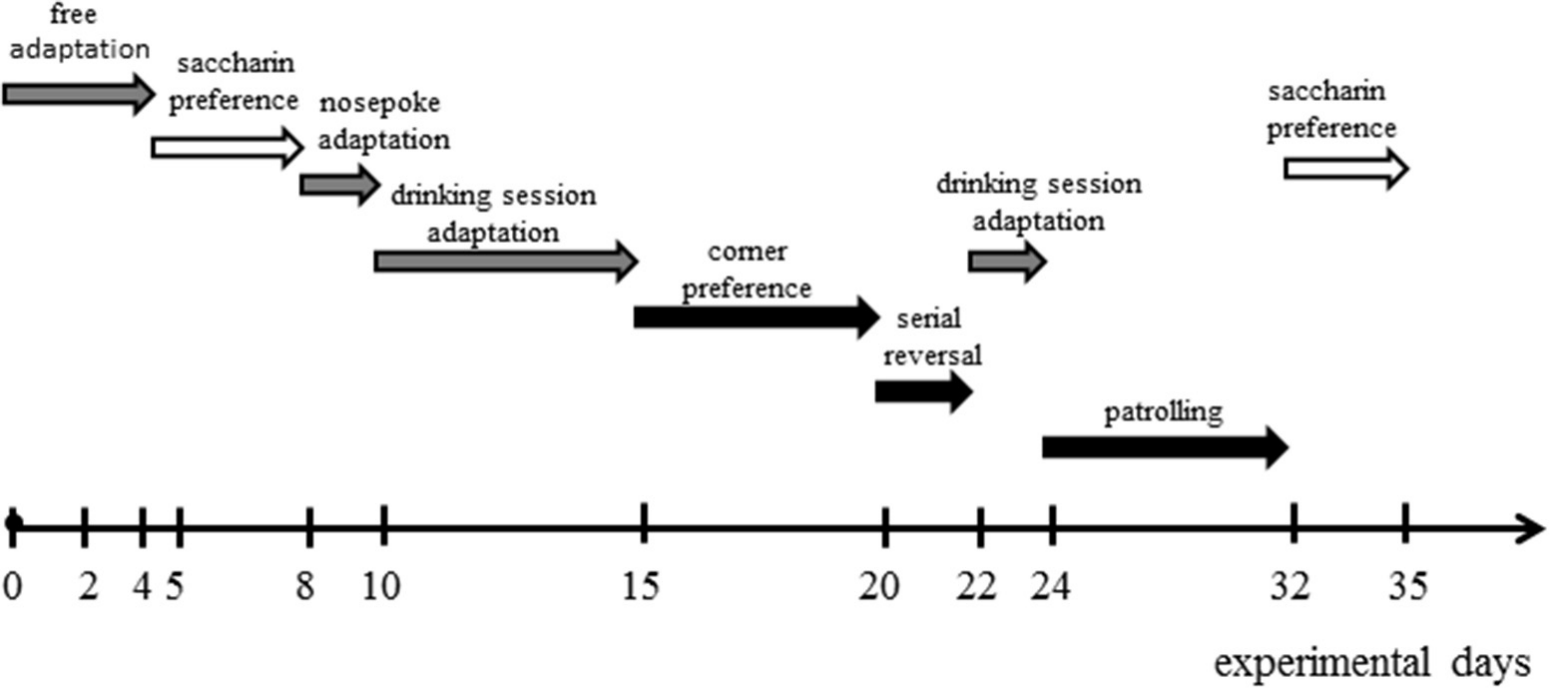
**Timeline of protocols in IntelliCage**. Gray arrows mark the adaptation sessions, black arrows—memory tests, white arrows—assessment of saccharin preference.

The monitoring in IntelliCage consisted of the adaptation sessions and learning protocols (corner preference, serial reversal and patrolling) (Kobayashi et al., 2013; Kulesskaya et al., 2013) applied in the order presented below and in Figure 1.

#### Free adaptation

During free adaptation, water was available in all corners *ad libitum*, doors to water were open. Duration and number of corner visits as well as number of licks were recorded for 2 days.

#### Extended adaptation

During the next two days, both doors in the chamber were opened for 7 s from the beginning of a visit. In order to get access to water again, animals had to re-enter the corner.

#### Saccharin preference

Spontaneous preference to sweet taste was applied for measuring anhedonia. In every corner, one bottle was filled with 0.5% saccharin (in two corners on left and in two corners on right side) and another bottle with water. Both doors in the corner were opened for 7 s from the beginning of visit, allowing the choice between two bottles. The number of licks from each bottle was recorded and preference for saccharin was calculated as a percentage from total number of licks. Anhedonia was measured twice: during days 5–8 and days 32–35 after beginning of the experiment.

#### Nosepoke adaptation

The first nosepoke of the visit opened the respective door for 7 s. In order to open the door again, animals had to re-enter the corner.

#### Drinking sessions (5 days, 10 sessions)

The mice were adapted to a fixed drinking schedule with doors opening in response to nosepokes (as in previous protocol) only between 8–10 p.m. and 4–6 a.m. The following learning tasks were carried out in drinking sessions.

#### Corner preference (5 days, 10 sessions)

The mice within the cage were divided into subgroups of 2–3 subjects and water was available only in one corner during drinking sessions. Assignment of the corner to individual mice was based on the visiting preference during the previous phase and the most and least preferred corners were excluded (average preference to the assigned corner was close to 25%, i.e., chance level).

#### Serial reversal (2 days, 4 sessions)

In a serial reversal task a “correct” (rewarded) corner was changed for the subgroup of 2–3 animals for every subsequent drinking session. In the first session, the corner was opposite to the previously learned one, then changed to the adjacent, opposite and adjacent again (e.g., if corner preference was carried out in corner 1, then the sequence for serial reversal was 3-4-2-1).

#### Patrolling (8 days, 16 sessions)

In patrolling protocol, the animals had to move from one corner to the next in a clockwise or anticlockwise direction in order to get access to water. The next rewarded corner was always adjacent to the corner most recently rewarded.

In all learning protocols, the visits with nosepokes were counted and the results were assessed as a percentage of the number of visits to the “correct” corner from the total number of visits to all corners.

### Individual behavioral tests

Classical behavioral tests were performed after the IntelliCage experiment. Animals were moved to the standard cages while being kept in the same social groups. The tests were done between 9 a.m. and 2 p.m. (during the light phase) with the interval of 2–3 days in the order presented below.

#### Light-dark test

Experimental arena (30 × 30 cm, MedAssiociates, St. Albans, VT) was divided by dark insert (non-transparent for visible light) into two equal parts: white open zone (illuminated at ~1000 lux) and dark zone. Light and dark areas were connected by opening at floor level (5.5 × 7 cm). The test was started by placing a mouse into the dark area and the animal was allowed to explore the experimental chamber freely for 10 min. The number of entries and the time spent in either zone as well as the number of rearing were detected by infrared sensors and collected by an activity monitor system (Version 5, MedAssiociates, St. Albans, VT).

#### Forced swim test

The mouse was placed in the glass cylinder (diameter 18 cm, height 25 cm) filled with tap water at room temperature (22 ± 1°C). The trials were recorded by video-tracking system (EthoVision XT 8.0, Noldus, The Netherlands) and time of immobility (passive floating) was measured during 6 min of the test.

#### Tube test

Tube test was used for assessment of social dominance/avoidance. Two unfamiliar animals were released simultaneously from the opposite ends of a transparent plastic tube (30 cm length, 3 cm inner diameter). The test ended when one of the animals was retreated from the tube. The test was not scored if both animals stayed in the tube for more than 2 min or crossed over each other. Every mouse was tested against all suitable unfamiliar mice from the opposed group. The percentage of the wins was calculated for every mouse. Test was done on two days. On the first day, social dominance was compared between the animals of different strains in the same housing condition: mixed BL6 vs. mixed DBA/2, separate BL6 vs. separate DBA/2. On the second day, the animals of the same strain from different housing conditions competed with each other: mixed BL6 vs. separate BL6, mixed DBA/2 vs. separate DBA/2.

### Messenger RNA analysis

A few days after the last behavior experiment, animals were killed by carbon dioxide, the hypothalamus, hippocampus and cortex were dissected, immediately frozen on liquid nitrogen and kept at −80°C. Total RNA was extracted using QIAzol Lysis reagent (Qiagen Nordic, Sweden) according to the manufacturer's instructions. 1 μg of total RNA was treated with DNAse I (Thermo Scientific, Finland) and reverse transcribed using oligo(dT) primer and a RevertAid First Strand cDNA Synthesis Kit (Thermo Scientific, Finland). The amount of transcripts was quantified using Maxima SYBR Green real-time PCR mix (Thermo Scientific, Finland) with primers specified in Table 1. DNA amplification reactions were run in triplicate. *Ct* values from each sample were obtained using the LightCycler 480 software. Relative quantification of template was performed using ΔΔ*Ct* method. Three “housekeeping” genes were considered for normalization of cDNA data (*Gapdh, Hprt* and *Rn18s*) and the analysis of the raw *Ct* values detected the effects of strain and/or housing on their expression. For *Hprt* and *Rn18s*, these effects were tissue- and/or housing-dependent whereas the *Gapdh* levels were affected only by the strain factor (higher in BL6 females) equally throughout all three tissues (Table 1). Thus, because the present study is focused on the analysis of the effect of housing, the cDNA data were normalized to the *Gapdh* level not affected by this factor. Control reactions without reverse transcriptase were also performed.

**Table 1:** **Primers used to amplify specific cDNA regions of the transcripts**.

**Gene**	**Forward**	**Reverse**
*Nr3c1*	CTGCCACAGCTTACCCCTAC	ATCCTGGTATCGCCTTTGCC
*Bdnf* total	GAAGGCTGCAGGGGCATAGACAAA	TACACAGGAAGTGTCTATCCTTATG
*Bdnf1*	CAAGACACATTACCTTCCTGCATCT	ACCGAAGTATGAAATAACCATAGTAAG
*Bdnf4*	TGTTTACTTTGACAAGTAGTGACTGAA	ACCGAAGTATGAAATAACCATAGTAAG
*Oxtr*	CTTCTTCGTGCAGATGTG	GAGCAGAGCAGCAGAGGAAG
*Avr1a*	CATCCTCTGCTGGACACCTT	TTCAAGGAAGCCAGTAACGC
*Gapdh*^a^	GGTGAAGGTCGGTGTGAACGG	CATGTAGTTGAGGTCAATGAAGGG
*Hprt*^b^	GGAAAGAATGTCTTGATTGTTGAAG	GTCCTTTTCACCAGCAAGCTTG
*Rn18s*^c^	CATGGCCGTTCTTAGTTGGT	GAACGCCACTTGTCCCTCTA

^a,b,c^Two-Way ANOVA analysis of the levels of housekeeping genes (based on the raw Ct values):

a *Strain effect: cortex F_(1, 24)_ = 81.29, p < 0.0001; hippocampus F_(1, 24)_ = 50.75, p < 0.0001; hypothalamus F_(1, 24)_ = 33.06, p < 0.0001*.

b *Cortex strain effect F_(1, 24)_ = 13.13, p < 0.01 and strain × housing interaction F_(1, 24)_ = 4.43, p < 0.05*.

c *Cortex strain effect F_(1, 24)_ = 32.15, p < 0.0001; hippocampus housing effect F_(1, 24)_ = 4.30, p < 0.05 and strain × housing interaction F_(1, 24)_ = 4.91, p < 0.05*.

### Serum corticosterone measurement

Blood was taken from the heart and placed at +4°C overnight. Blood serum was retrieved by centrifugation at 5000 rpm for 25 min. Corticosterone level was measured by a corticosterone enzyme immunoassay kit (Arbor Assays, US) according to the manufacturer's instructions.

### Statistical analyses

Nonparametric Mann-Whitney test was used for analyses of data in tube test. Other data were analyzed by factorial ANOVA with strain (BL6 and DBA/2) and housing condition (separate and mixed) as independent variables followed by *post-hoc* Tukey test for detection of group differences. Data are presented as means and standard errors on all figures.

## Results

All results of behavioral tests are summarized in Table 2. During the whole experiment, DBA/2 mice had higher body weight without dependence on social grouping.

**Table 2:** **Behavior in IntelliCage and individual tests**.

**Parameters**	**Strain (BL6 vs. DBA/2)**	**Housing (mixed vs. separate)**	**Strain × housing interaction**
	***F*_(1, 52)_, *p*-value**	***F*_(1, 52)_, *p*-value**	***F*_(1, 52)_, *p*-value**
**BODY WEIGHT, g**			
Body weight, day 0	37.619; <0.0001 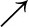	ns	ns
Body weight, day 38	35.118; <0.0001 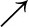	ns	ns
**IntelliCage, FREE ADAPTATION**			
Visits in first 10 min, no	ns	ns	ns
Visits in first 30 min, no	ns	ns	ns
Visits in first 60 min, no	ns	ns	ns
Visits in first 8 h, no	9.484; <0.01 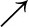	ns	4.249; <0.05
Visits in light, no	152.279; <0.0001 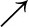	ns	ns
Visits in dark, no	ns	ns	ns
Visit duration in light, s	7.376; <0.01 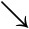	11.706; <0.01 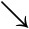	7.798; <0.01
Visit duration in dark, s	30.358; <0.0001 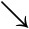	ns	ns
Licks in light, no	136.475; <0.0001 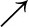	5.757; <0.05 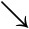	6.747; <0.05
Licks in dark, no	61.168; <0.0001 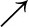	ns	ns
**IntelliCage, CORNER PREFERENCE**			
Correct corners visits, aver %	28.089; <0.0001 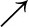	ns	ns
Coner visits, *n*	52.369; <0.0001 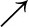	ns	ns
**SERIAL REVERSAL IN IntelliCage**			
Correct corners visits, aver %	12.829; <0.001 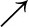	ns	ns
Corner visits, *n*	20.139; <0.0001 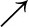	ns	ns
**IntelliCage, PATROLLING**			
Correct corners visits, aver %	5.772; <0.05 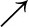	8.883; <0.01 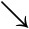	ns
Corner visits, *n*	26.568; <0.0001 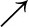	ns	ns
**IntelliCage, SACCHARIN PREFERENCE**			
Saccharin preference (day 5–8), %	ns	ns	ns
Saccharin preference (day 35–38), %	ns	11.128; <0.01 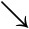	11.238; <0.01
	***F*_(1, 24)_, *p*-value**	***F*_(1, 24)_, *p*-value**	***F*_(1, 24)_, *p*-value**
**LIGHT-DARK TEST**			
Time in light, s	40.125; <0.0001 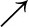	7.788; <0.05 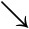	ns
Distance in light, %	30.709; <0.0001 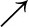	ns	ns
Distance, cm	42.921; <0.0001 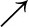	12.096; <0.01 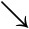	ns
Latency to first light entry, s	ns	ns	ns
Number of zone transitions, *n*	ns	ns	ns
Number of rearings, *n*	18.575; <0.001 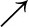	4.969; <0.05 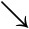	ns
**FORCED SWIM TEST**			
Immobility time, s	30.349; <0.0001	ns	ns

*Data were analyzed by Two-Way ANOVA. Arrows indicate comparisons (higher or lower value) of measured parameters in BL6 mice vs. DBA/2 or in mixed group vs. separate*.

### Housing in a mixed group differently affects spontaneous behavior of BL6 and DBA/2 mice in intellicage

**Figure 2:**
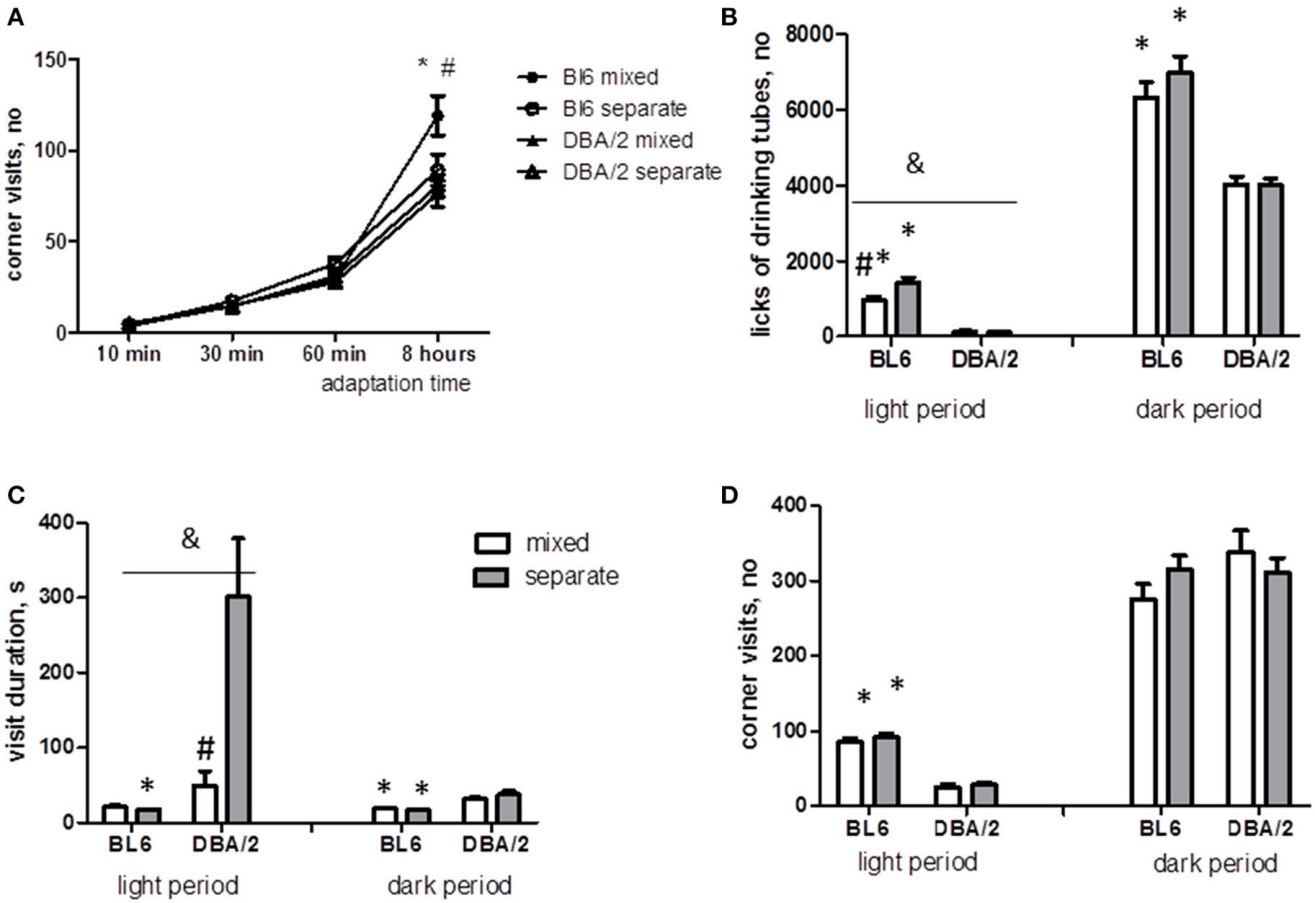
**Spontaneous behavior of mice in IntelliCage during adaptation period**. **(A)** The cumulative number of visits to operant chambers during first 8 h of adaptation to IntelliCage and new social group is significantly higher in BL6 mixed mice than in other groups. **(B)** BL6 mice of both housing groups perform an increased number of licks than DBA/2 mice. Mixed housing increases the number of licks in BL6 mice but not in DBA/2 mice during light period. **(C)** The average duration of visits to corner chamber is higher in DBA/2 mice than BL6 mice. Mixed housing dramatically decreases the duration of visits in DBA/2 mice during light period. **(D)** The number of visits to corner chambers during light period is higher in BL6 mice than in DBA/2 and does not depend on housing conditions. ^*^p < 0.05 in Tukey *post-hoc* test for comparison of BL6 with DBA/2 mice from the same housing group; ^#^*p* < 0.05 in Tukey *post-hoc* test for comparison of mixed with separated animals of the same strain; ^&^*p* < 0.05 Two-Way ANOVA test for interaction of “strain” × “housing” factors (B6-separate *n* = 16, D2-separate *n* = 16, B6-mixed *n* = 12, D2-mixed *n* = 12).

Spontaneous behavior in IntelliCage was measured during the first two days of free adaptation period. After the first 8 h of adaptation to new environment, mixed housing significantly increased the number of visits to operant chambers in BL6 mice but did not affect DBA/2 animals (strain × housing interaction *p* < 0.05, Figure 2A). During the next 2 days, DBA/2 animals displayed lower number of corner visits (*p* < 0.0001, Figure 2D) and licks (*p* < 0.0001 for light period, *p* < 0.0001 for dark period, Figure 2B), whereas the duration of corner visits was longer than in BL6 mice (*p* < 0.01 light period, *p* < 0.0001 dark period, Figure 2C). Some of the DBA/2 mice in separated cages were observed as sleeping in operant chambers during the light period. Therefore, the visits longer than 1000s were excluded from the analysis of visit duration. The effect of housing on visit duration and lick number during the light period was revealed by significant interaction between the strain and housing condition (lick number *p* < 0.05, visit duration *p* < 0.05). *Post-hoc* comparison showed that mixed housing shortened the visit duration in DBA/2 mice and reduced the number of licks in BL6 mice (Figures 2B,C).

### Housing in a mixed group impairs learning abilities of BL6 mice and does not affect DBA/2 mice in intellicage

**Figure 3:**
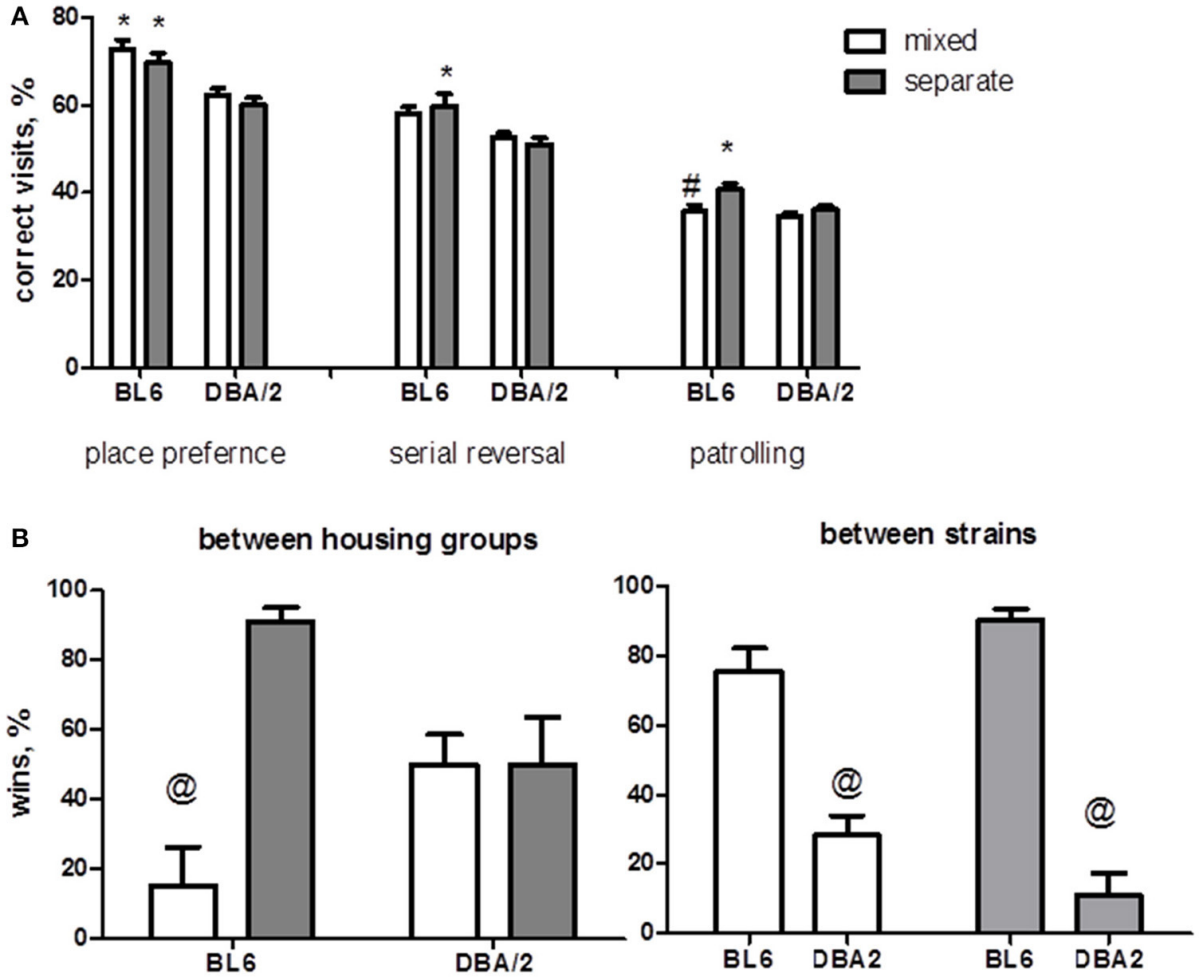
**Cognitive and social behavior**. **(A)** DBA/2 mice demonstrate cognitive deficit in all three learning paradigms applied in the IntelliCage (shown as a reduction in the percentage of correct visits). Mixed housing results in a decline of learning ability of BL6 mice in patrolling task, while the learning efficiency of DBA/2 animals does not depend on housing factor. **(B)** DBA/2 mice display social withdrawal demonstrating a lower percentage of wins in competition between strains in the tube test of social dominance. Mixed BL6 animals winless trials in competition with BL6 from a separate group, while housing factor is not effective in DBA/2 animals. ^*^p < 0.05 in Tukey *post-hoc* test for comparison of Bl6 with DBA2 mice from the same housing group; ^#^*p* < 0.05 in Tukey *post-hoc* test for comparison of mixed with separated animals of the same strain, ^@^*p* < 0.05 in Mann-Whitney test (B6-separate *n* = 16, D2-separate *n* = 16, B6-mixed *n* = 12, D2-mixed *n* = 12).

Several studies have demonstrated the difference in cognitive functions of DBA/2 and BL6 mouse strains (Crawley et al., 1997; Voikar et al., 2005). Therefore, we were interested in possible effect of social conditions on learning in the home cages. Predictably, BL6 mice were more effective in corner preference (*p* < 0.0001), serial reversal (*p* < 0.001) and patrolling tasks (*p* < 0.05) (Figure 3A, Table 2). However, *post-hoc* comparisons showed that the BL6 mice from the mixed group had less “correct” visits in patrolling task than BL6 mice from the separate group. Thus, housing in a mixed group significantly impaired patrolling task performance in BL6 animals and did not affect behavior of DBA/2 mice.

### DBA/2 mice demonstrate social withdrawal and affect social behavior of BL6 mice

Our preliminary experiments have revealed deficient nest building in the DBA/2 mice as compared to BL6 (data not shown). Nesting behavior has been proposed as an indicator of social communication (Crawley, 2004) and nest formation is used to measure autism-like behaviors in mice (Satoh et al., 2011). Therefore, we performed a tube test to measure social dominance/ avoidance between the animals of different strains and housing groups. The tube test was done in two versions: competition between DBA/2 and BL6 mice of the same housing group and between mixed and separate animals of the same strain. In the inter-strain competition, BL6 mice won more trials than DBA/2 mice (*p* < 0.0001 mixed group, *p* < 0.001 separate group). However, comparison of the housing groups revealed that mixed housing reduced significantly the percentage of wins in BL6 (*p* < 0.01), but had not effect in DBA/2 (Figure 3B).

### Mixed housing increases anhedonia and anxiety-like behavior in BL6 mice but does not affect despair-like behavior

**Figure 4:**
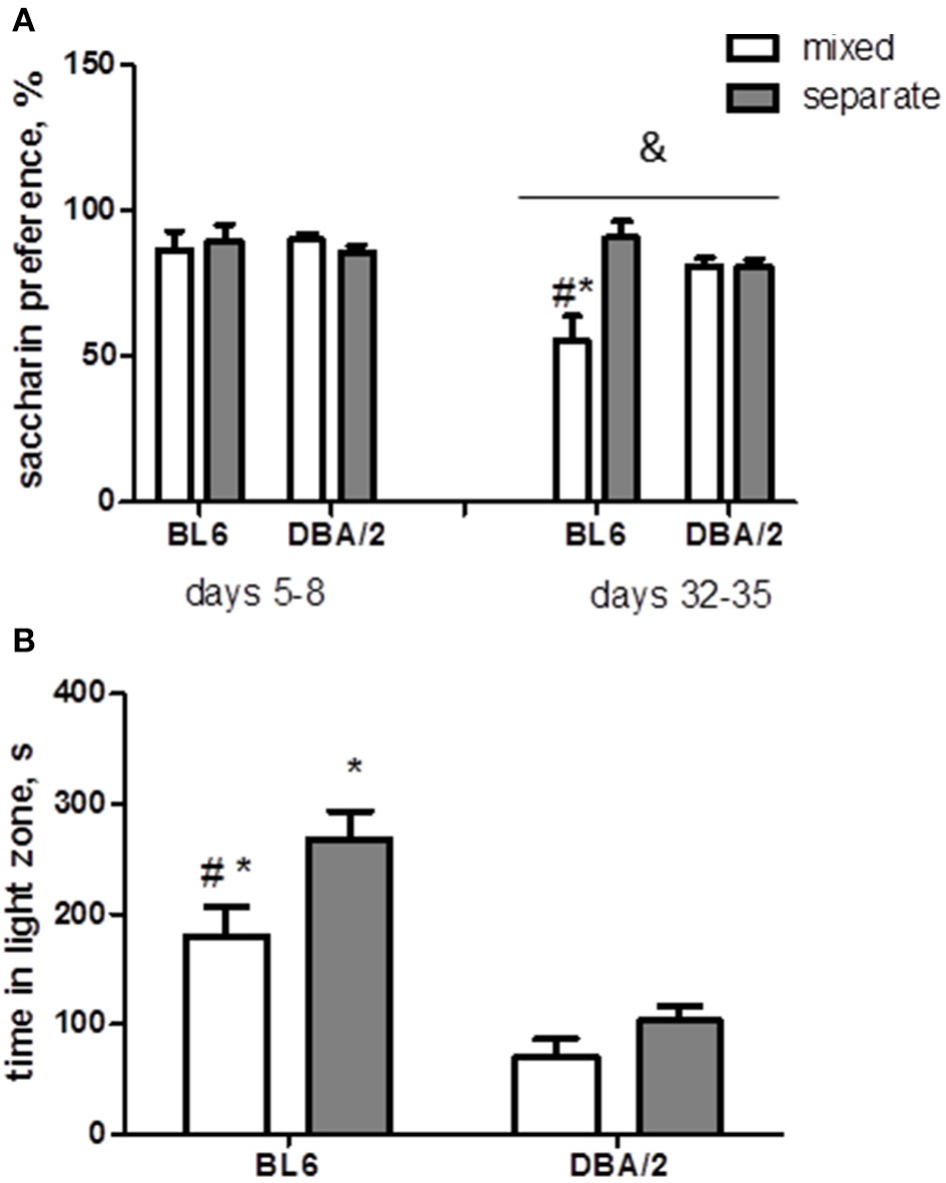
**Emotional behavior**. **(A)** There is no difference in saccharin preference during the experimental days 5–8; on the days 32–35, the percentage of saccharin consumption is significantly reduced in BL6 mixed animals in comparison with separate animals from the same strain and DBA/2 mice. **(B)** During a light-dark test BL6 mice spend more time in the light zone than DBA/2 mice; mixed housing had a significant effect on behavior of BL6 mice reducing the period they spend in the light compartment. ^*^p < 0.05 in Tukey *post-hoc* test for comparison of BL6 with DBA/2 mice from the same housing group; ^#^*p* < 0.05 in Tukey *post-hoc* test for comparison of mixed with separated animals of the same strain; ^&^*p* < 0.05 Two-Way ANOVA test for interaction of “strain” × “housing” factors (B6-separate *n* = 8, D2-separate *n* = 8, B6-mixed *n* = 6, D2-mixed *n* = 6).

To explore the emotional condition of the mice housed in the mixed group, we assessed anhedonia, anxiety-like behavior and behavioral despair. Anhedonia, or loss of interest to pleasure, is one of the symptoms of several neuropsychiatric disorders including schizophrenia and depression (Der-Avakian and Markou, 2012). Measurement of preference to sweet taste is a method of assessing anhedonia in animals. Saccharin consumption was similar for all groups on days 5–8 after the beginning of the experiment and was decreased significantly in BL6 mice in mixed housing as compared to the other groups on days 32–35 (strain x housing interaction *p* < 0.01, Figure 4A).

Light-dark test is widely used to assess anxiety in mice. The BL6 animals demonstrated reduced anxiety-like behavior compared to DBA/2 mice as suggested by increased number of rearing (*p* < 0.001), increased proportion of distance (*p* < 0.0001) and time (*p* < 0.0001) in the light compartment (Figure 4B). Mixed housing significantly altered the anxiety level only in BL6 mice and did not affect DBA/2 mice (Figure 4B): the time spent and the percent of distance traveled in the light zone were reduced in BL6 mixed mice in comparison with BL6 separate animals.

The behavioral despair in inescapable situations was measured as an immobility time in a forced swim test. The BL6 mice demonstrated longer immobility time (*p* < 0.0001) in comparison with DBA/2 mice without any effect of housing (Table 2).

Altogether, housing conditions did not affect DBA/2 mice, but resulted in increased anxiety and anhedonia in BL6 mice co-housed with DBA/2 animals.

### Mixed housing increases serum corticosterone in both strains

**Figure 5:**
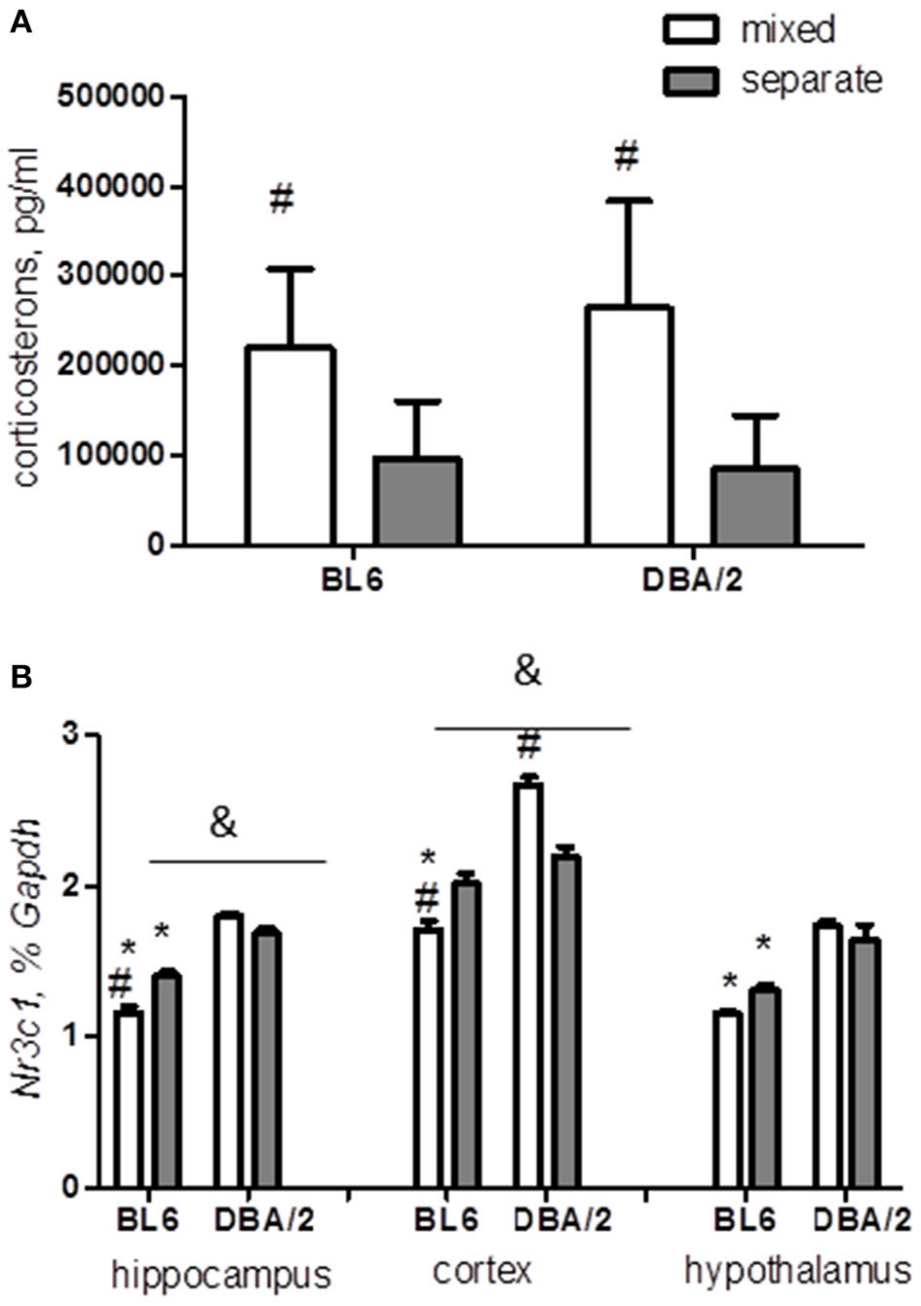
**Corticosterone content and expression of glucocorticoid receptor *Nr3c1***. **(A)** Mixed housing results in an increase of serum corticosterone level in mice of both strains in comparison with separate-housed groups. **(B)** The strain difference in glucocorticoid receptor expression is determined by the effects of mixed housing on the hippocampus and cortex. ^*^p < 0.05 in Tukey *post-hoc* test for comparison of BL6 with DBA/2 mice from the same housing group; ^#^*p* < 0.05 in Tukey *post-hoc* test for comparison of mixed with separated animals of the same strain; ^&^*p* < 0.05 Two-Way ANOVA test for interaction of “strain” × “housing” factors (B6-separate *n* = 8, D2-separate *n* = 8, B6-mixed *n* = 6, D2-mixed *n* = 6).

The mixed housing increased significantly the serum levels of corticosterone in both mouse strains in comparison with the separate housing (*p* < 0.0001, Figure 5A). This result suggests that housing in a mixed group might represent a stressful environment for females of both BL6 and DBA/2 strains.

### Mixed housing induces opposite changes in glucocorticoid receptor expression in BL6 and DBA/2 mice

We next investigated whether the expression of the glucocorticoid receptor encoded by the gene *Nr3c1* (Boyle et al., 2005; McGowan et al., 2009) was affected by the mixed housing. The effect of housing was determined by the strain factor in both the hippocampus and the cortex (strain × housing interaction: hippocampus *p* < 0.0001; cortex *p* < 0.0001). We found that the hippocampal and cortical *Nr3c1* expression was significantly down-regulated by the mixed housing in BL6 mice, with a similar trend in the hypothalamus (Table 3, Figure 5B). Unexpectedly, DBA/2 mice in the mixed cages showed enhanced *Nr3c1* expression in the cortex in comparison with the separated mice.

**Table 3:** **Corticosterone content and expression of several stress-related genes**.

**Parameters**	**Strain (BL6 vs. DBA/2)**	**Housing (mixed vs. separate)**	**Strain × housing interaction**
Corticosterons in serum, pg/ml	ns	23.201; <0.0001 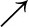	ns
**Hippocampus**	***F*_(1, 24)_, *p*-value**	***F*_(1, 24)_, *p*-value**	***F*_(1, 24)_, *p*-value**
BDNF1, % gapdh	8.416; <0.01 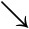	10.747; <0.01 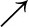	16.871; <0.001
BDNF4, % gapdh	ns	ns	20.829; <0.0001
BDNF total, % gapdh	7.505; <0.05 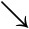	ns	4.934; <0.05
Glucocorticoid receptor 1, % gapdh	160.583; <0.0001 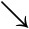	ns	24.051; <0.0001
Arginine vasopressine RA1 mRNA, % gapdh	ns	ns	ns
Oxytocine R mRNA, % gapdh	13.065; <0.01 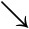	ns	ns
**CORTEX**			
BDNF1, % gapdh	4.949; <0.05 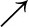	ns	32.003; <0.0001
BDNF4, % gapdh	ns	ns	6.039; <0.05
BDNF total, % gapdh	ns	ns	16.168; <0.001
Glucocorticoid receptor 1, % gapdh	71.687; <0.0001 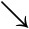	ns	33.693; <0.0001
Arginine vasopressine RA1 mRNA, % gapdh	Ns	ns	10.132, <0.01
Oxytocine R mRNA, % gapdh	Ns	ns	7.091; <0.05
**HYPOTHALAMUS**			
BDNF1, % gapdh	21.524; <0.0001 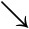	ns	ns
BDNF4, % gapdh	7.351; <0.05 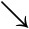	ns	ns
BDNF total, % gapdh	34.907; <0.0001 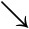	ns	ns
Glucocorticoid receptor 1, % gapdh	46.044; <0.0001 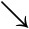	ns	ns
Arginine vasopressine RA1 mRNA, % gapdh	6.680; <0.05 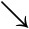	ns	ns
Oxytocine R mRNA, % gapdh	17.093; <0.001 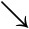	ns	ns

*The data were analyzed by Two-Way ANOVA. Arrows indicate comparisons (higher or lower value) of measured parameters in BL6 mice vs. DBA/2 or in mixed group vs. separate*.

### Differential effects of housing on *Bdnf* expression in BL6 and DBA/2 mice

**Figure 6:**
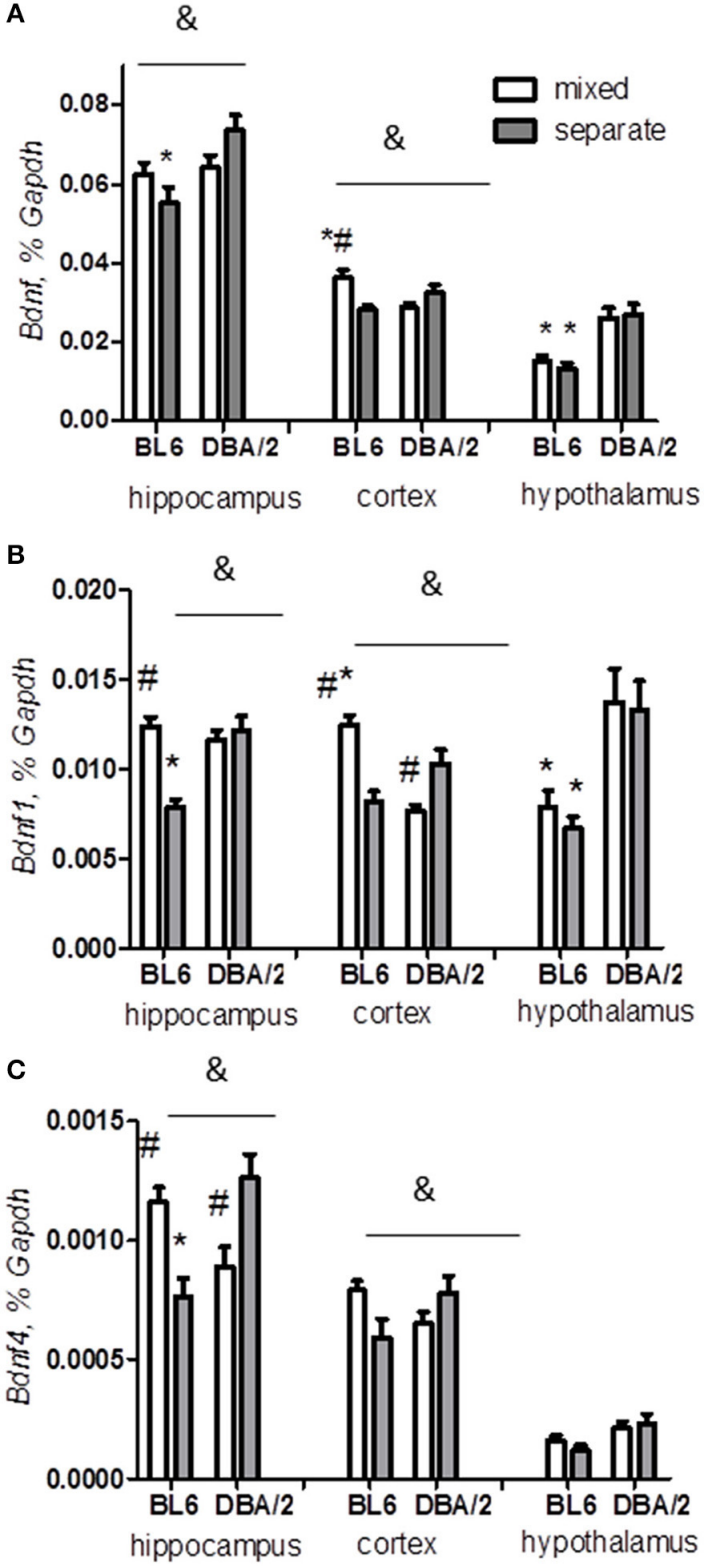
**Expression of *Bdnf* transcripts**. The effect of housing condition to total amount of *Bdnf* transcripts **(A)** and activity-dependent individual transcripts *Bdnf1* **(B)** and *Bdnf4* **(C)** is significantly affected by the strain factor in the hippocampus and cortex: mixed housing increases the expressions of these transcripts in BL6 and decreases them in DBA/2 mice. ^*^p < 0.05 in Tukey *post-hoc* test for comparison of BL6 with DBA/2 mice from the same housing group; ^#^*p* < 0.05 in Tukey *post-hoc* test for comparison of mixed with separated animals of the same strain; ^&^*p* < 0.05 Two-Way ANOVA test for interaction of “strain” × “housing” factors (B6-separate *n* = 8, D2-separate *n* = 8, B6-mixed *n* = 6, D2-mixed *n* = 6).

A key neuronal plasticity-related gene, brain-derived neurotrophic factor, *Bdnf*, is known to be actively regulated by stress (Nestler et al., 2002; Aid et al., 2007; Kundakovic et al., 2013; Karpova, 2014). We were particularly interested in whether different housings had an effect on the total level of *Bdnf* mRNA and on the activity-dependent *Bdnf* transcripts 1 and 4. We found that the housing factor affected both hippocampal and cortical *Bdnf* levels, showing opposite changes in BL6 and DBA/2 mice (Figures 6A–C, Table 3) (strain x housing interaction: hippocampal total *Bdnf p* < 0.05, *Bdnf1 p* < 0.001, *Bdnf4 p* < 0.0001; cortical total *Bdnf p* < 0.001, *Bdnf1 p* < 0.0001, *Bdnf4 p* < 0.05): while the mixed housing increased the expression of *Bdnf* transcripts in BL6 mice, it decreased the *Bdnf* levels in DBA/2 mice. In the hypothalamus, housing conditions did not affect *Bdnf* levels (Figures 6A–C, Table 3).

### Expressions of arginine vasopressin receptor and oxytocin receptor in the cortex are strain- and housing-dependent

**Figure 7:**
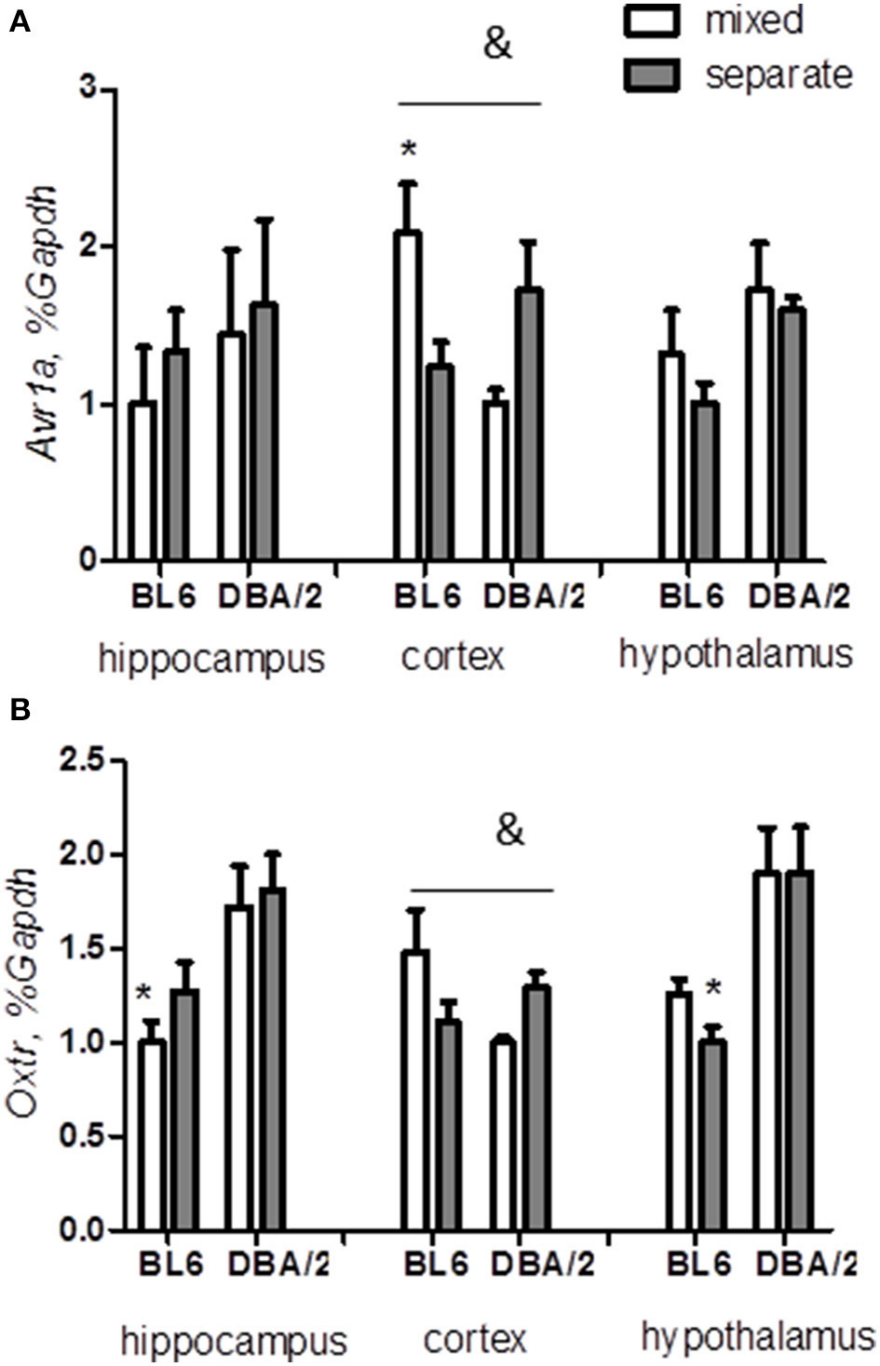
**Expression of arginine vasopressin receptor A1 and oxytocin receptor**. The effect of housing condition on expressions of *Avr1a* **(A)** and *Oxtr* **(B)** is modulated by the strain factor in the cortex. ^*^p < 0.05 in Tukey *post-hoc* test for comparison of BL6 with DBA/2 mice from the same housing group; ^#^*p* < 0.05 in Tukey *post-hoc* test for comparison of mixed with separated animals of the same strain; ^&^*p* < 0.05 Two-Way ANOVA test for interaction of “strain” × “housing” factors (B6-separate *n* = 8, D2-separate *n* = 8, B6-mixed *n* = 6, D2-mixed *n* = 6).

Arginine vasopressin and oxytocin are neuropeptides that contribute to both social behaviors and stress reactivity in mammalians (Jezova et al., 1995; Insel, 2010; Ebstein et al., 2012; Benarroch, 2013; Lukas and Neumann, 2013). We measured the expressions of the receptors for these hormones in mixed and separated BL6 and DBA/2 mice. The regulatory effect of housing condition on either the arginine vasopressin receptor 1 (*Avr1a*) (strain x housing interaction *p* < 0.01) or the oxytocin receptor (*Oxtr*) (strain x housing interaction *p* < 0.05) was strain-dependent in the cortex (Figures 7A,B, Table 3). Housing condition did not affect the expression of these receptors in the hypothalamus and hippocampus, however.

## Discussion

In the present work we were interested in the modulatory effect of social context on home-cage behavior, learning abilities, emotional conditions, and social activity in group-housed female mice. Depending on the context, consequences of social experience could have negative or positive effect resulting in the development of stress or providing an enriched environment and social protection. Social stress seems to be critical for the development of several neuropsychiatric disorders such as schizophrenia, depression, and posttraumatic stress disorders (Kessler et al., 1985; Van Os and Mcguffin, 2003; Cantor-Graae, 2007; Pulkki-Raback et al., 2012). On the other hand, social support and social learning can improve or even prevent the development of some behavioral pathologies. Animal models are widely used to investigate the mechanisms of neuropsychiatric disorders and to validate the strategies for their treatment. Mixing of mice with different genotypes and phenotypes could be used as a model of behavioral intervention based on social enrichment for the correction of various behavioral abnormalities. For instance, co-housing of BL6 and BTBR [a mouse model of autism-like behavior (Moy et al., 2007)] strains has been effective in rescuing the social deficits in the BTBR strain (Yang et al., 2011). Another study has shown a beneficial effect of co-housing the wild type and transgenic mice on learning performance in a model of Alzheimer's disease (Kiryk et al., 2011). Social learning is involved in the transmission of social behaviors, which has been described not only for humans but also for animals. In rodents, the effectiveness of observational learning has been shown in multiple learning paradigms. For instance, Lipina and Roder have recently demonstrated that paired co-learning (simultaneous involvement to the same activity) with a familiar BL6 mouse rescued the deficit in episodic memory in BTBR mice. Co-learning reduced the anxiety level, increased the exploratory behavior and mediated the learning-facilitation in a novel environment (Lipina and Roder, 2013). Therefore, our expectation of the positive effect would be reduced anxiety-like behavior and improved learning in DBA/2 mice when BL6 and DBA/2 strains were mixed.

### Co-housing with BL6 mice does not improve cognitive function and social behavior in DBA/2 mice

In concordance with our expectations and literature (Crawley et al., 1997; Bouwknecht and Paylor, 2002; Voikar et al., 2005; Moy et al., 2007), we found increased anxiety level and decreased learning abilities in DBA/2 mice as compared to BL6 mice. Surprisingly, very few comparative studies on social behavior in these strains exist. It has been shown that BL6 and DBA/2 mice differ in maternal behavior (Brown et al., 1999) and DBA/2 male mice display decreased sociability in the conventional 3-chamber test (Moy et al., 2008). On the other hand, other papers have reported the absence of difference in 3-compartment test (Moy et al., 2007) and in the social transmission of food preference (Holmes et al., 2002). In addition, female DBA/2 mice showed reduced nest building (our unpublished data).

Based on previous works, we proposed that socially active BL6 mice could engage their cage-mates to a more active interaction and provide an opportunity for observational learning in cognitive tasks. However, our results did not reveal any positive alteration in cognitive functions or social behavior in tube test in the mixed DBA/2 mice as compared to the separate ones. It is important to consider the age of the animals at the time of manipulations with social environment. Most likely, adolescent, and adult mice, as well as male and female mice react differently on the applied social interventions. The social instability paradigm has been well validated with the adolescent mice (Schmidt et al., 2007, 2010). Moreover, postnatal environment can have a profound effect on later response to social stress (Branchi et al., 2010, 2013). Yang with colleagues demonstrated that a peer-enrichment strategy started immediately after weaning improved significantly social deficit in BTBR adolescent mice (Yang et al., 2011). Our experiment was carried out in adult female mice and the only finding that could be described as an improvement of social behavior was a decrease of time that DBA/2 mice spent in corner chambers of an IntelliCage (duration of corner visit) during the light period. Mice and rats are nocturnal animals and the inactive period they spend mostly in sleeping in huddle with cage-mates (Tulogdi et al., 2014). The tendency of DBA/2 mice to occupy operant chambers and sleep there alone during the light period can be interpreted as social avoidance and escape to isolated shelters. Indeed, the mixed housing with BL6 mice resulted in a significant reduction of the duration of corner visit in DBA/2 mice. However, the design of this study did not allow us to recognize whether this reduction was voluntary or forced by BL6 animals or whether it reflected some improvement of social function. To answer this question, further validation of mixed housing paradigm in an IntelliCage with additional extensions (such as separate social boxes) and more detailed exploring of huddling behavior during sleeping are needed.

Theoretically the number of individuals in the cage could have an effect on the behavioral phenotype. This has been consistently demonstrated in the studies assessing the effects of crowding, isolation, or social instability. However, our aim was to keep the mice in stable social groups without applying any of the aforementioned stressful changes in social conditions. In order to control the effect of the number of individuals housed together, further experiments involving groups differing only in the strain and not in the number of individuals should be performed.

### Mixed housing with DBA/2 mice negatively affects multiple aspects of behaviors in BL6 females

The most interesting result of our experiments was that BL6 animals were more sensitive to the applied behavioral implementation than DBA/2 mice. BL6 mice showed distinct signs of anhedonia (reduced saccharin preference), learning impairment (patrolling in the IntelliCage), and increased anxiety (light-dark test) and social avoidance (tube test). Altered anxiety level and social behavior as well as disturbance of reward system and impairment of cognitive functions are often described as behavioral consequences of stress exposure (Blanchard et al., 2001; Haller et al., 2004; Buwalda et al., 2005; Campeau et al., 2011; Hill et al., 2012). BL6 and DBA/2 mice have been shown to react differently to stressful manipulations of different nature, which could be explained by different coping styles (active vs. passive) exhibited by these strains. For instance, DBA/2 mice displayed increased anxiety-like behavior after chronic immobilization (Mozhui et al., 2010) and long-term individual housing (Voikar et al., 2005). In addition, DBA/2 mice were more susceptible to stress in social defeat paradigm (Sokolowska et al., 2013). On the other hand, DBA/2 mice performed better than BL6 mice in the one-way avoidance task (Weinberger et al., 1992), suggesting the exploitation of active coping style. Moreover, BL6 mice showed enhanced immobility in a forced swim test suggesting the use of passive coping style and susceptibility to stress-induced behavioral despair and depression-like behavior (Cabib et al., 2002, 2012; Voikar et al., 2005). Findings from the learned helplessness paradigm provide further support for such susceptibility in the BL6 mice (Shanks and Anisman, 1988). Therefore, behavioral manifestation of stress in BL6 and DBA/2 mice depends on the nature and duration of the stress and involves different genes (Cabib et al., 2002; Mozhui et al., 2010). Interestingly, a recent study suggested co-housing of BL6 and DBA/2 mice for facilitated individual identification (Walker et al., 2013) without any change in their behavior. However, another comparison of mixed housing of 129 (an “anxious” strain) and C57BL/6 mice has shown changes in anxiety-like behavior in both strains (Curley et al., 2010). Similar to our results, co-housing with 129 mice resulted in a trend to increased anxiety in BL6 mice.

In psychological literature, growing amount of evidence highlights a beneficial effect of family- and sibling-relationships in treatment of mental illnesses, but only a few studies investigated the impact of psychotic episodes, autism or mental retardation of affected persons on their healthy siblings (O'Brien et al., 2009; Bowman et al., 2014). Some groups have found a higher risk for social and cognitive deficits in siblings of children with autism (Shaked et al., 2006; Gamliel et al., 2009) and increased occurrence of psychotic diseases among previously healthy siblings of the patients with schizophrenia in comparison with siblings of typically developing children (Arajarvi et al., 2006). Noticeably, caregivers and spouses of patients with bipolar disorders and dementia often report depressive symptoms and use of mental service by themselves (Steele et al., 2010; Sadowsky and Galvin, 2012). Long-term interaction with affected subjects could result in distress and behavioral alterations in their “healthy” relatives and caretakers. Such kind of negative social experiences, as well as genetic influences, provide directions for further investigation of etiology of psychopathologies. The coping style could be one of the mediating mechanisms responsible for stress resilience both in humans (Steele et al., 2010) and animals. Active (problem-focused) coping style, including seeking social support, adopting a fighting spirit, reframing stressors in a positive light is effective in improvement of well-being and reduction of psychological symptoms in humans dealing with stressful life situation (Southwick et al., 2005). On the other hand, passive (emotion-focused) coping style has been associated with subjective burden and depression symptoms in family members and caregivers of patients with subarachnoid hemorrhage (Boerboom et al., 2014) and schizophrenia (Magliano et al., 2000), and older relatives (Del-Pino-Casado et al., 2011).

### Social context affects differently the neurobiology of C57BL/6 and DBA/2 mice

Our biochemical analysis suggests that DBA/2 and BL6 animals recruit different mechanisms for stress response. Firstly, we found that the corticosterone level was increased by the mixed housing in both strains, however, the expression of the glucocorticoid receptor showed opposite changes in mixed BL6 and DBA/2 mice. Glucocorticoid receptors provide a main inhibitory feedback in the HPA axis to reduce stress response and to decrease production of glucocorticoids (Sapolsky et al., 1985; Boyle et al., 2005). Reduced expression of glucocorticoid receptors is often associated with negative life experience, especially during childhood, and with increased stress susceptibility and risk of developing different psychopathologies (Sapolsky et al., 1985; Chourbaji et al., 2008; McGowan et al., 2009). Thus, an increased expression of glucocorticoid receptor induced by the mixed housing in DBA/2 mice could have a potential beneficial effect by normalizing their HPA function (Zhang et al., 2013b), and, consequently, their behavioral performance. Indeed, in contrast to BL6 mice, mixed housing of DBA/2 mice did not exacerbate their social and emotional behaviors, even though improvement was neither observed.

Secondly, Bdnf is another major regulator of the HPA axis and a key mediator of neuronal activity (Nestler et al., 2002; Castren et al., 2007; Karpova et al., 2009; Naert et al., 2011; Kundakovic et al., 2013; Karpova, 2014). Although chronic Bdnf deficiency was shown to impair learning and memory, Bdnf overexpression could mediate abnormal stress response (Soliman et al., 2010; Zhang et al., 2013a). Upregulated neuronal activity-dependent *Bdnf* transcripts were implicated in psychopathology of anxiety and vulnerability to addiction (Lubin et al., 2008; Wang et al., 2013). Consistent with these findings, we showed that the increase of *Bdnf* in BL6 mixed mice was accompanied by multiple stress–related behavioral impairments (increased anxiety, anhedonia, reduced cognitive and social functions). In agreement, impaired working memory and increased anxiety-like traits were revealed in females of Bdnf overexpressing mice (Papaleo et al., 2011).

Finally, we found modulatory effects of the mixed housing on the expression of receptors for oxytocin and arginine vasopressin in the cortex, in a similar pattern as observed for *Bdnf* expression. The role of these hormones in stress response, social behavior, and cognition (Benarroch, 2013; Lukas and Neumann, 2013) makes them interesting targets for a further exploration of their regulatory potential in social-related dysfunctions under our paradigm in the future.

## Conclusions

Our findings clearly highlight the importance of social factors in modulating the behavioral phenotypes of mice and call for cautious interpretations of behavioral changes incurred by this factor. Housing of animals with different behavioral phenotypes in a mixed group can severely affect the cognitive abilities, social functions, and emotional conditions. Mixed housing has been suggested to be applied as an animal model of behavioral intervention based on social enrichment in treatment of behavioral abnormalities. The results of our study draw attention on the possible negative impact of such interventions on individuals providing social enrichment and social learning.

## Author contributions

All authors participated in critical revising and final approval of the manuscript, and agree to be accountable for all aspects of the work. Natalia Kulesskaya and Nina N. Karpova interpreted data and drafted the manuscript, Li Tian and Vootele Voikar participated in writing the manuscript, Natalia Kulesskaya and Vootele Voikar were responsible for concept, design, data acquisition and analysis in behavioral part of work, Li Ma, Li Tian, and Nina N. Karpova designed and acquired data in biochemical and molecular experiments.

### Conflict of interest statement

The authors declare that the research was conducted in the absence of any commercial or financial relationships that could be construed as a potential conflict of interest.

## References

[B1] Aid T. Kazantseva A. Piirsoo M. Palm K. Timmusk T. (2007). Mouse and rat BDNF gene structure and expression revisited. J. Neurosci. Res. 85, 525–535 10.1002/jnr.21139 17149751 PMC1878509

[B2] Arajarvi R. Ukkola J. Haukka J. Suvisaari J. Hintikka J. Partonen T. (2006). Psychosis among “healthy” siblings of schizophrenia patients. BMC Psychiatry 6:6 10.1186/1471-244X-6-6 16448569 PMC1434733

[B3] Benarroch E. E. (2013). Oxytocin and vasopressin: social neuropeptides with complex neuromodulatory functions. Neurology 80, 1521–1528 10.1212/WNL.0b013e31828cfb15 23589638

[B5] Blanchard R. J. McKittrick C. R. Blanchard D. C. (2001). Animal models of social stress: effects on behavior and brain neurochemical systems. Physiol. Behav. 73, 261–271 10.1016/S0031-9384(01)00449-8 11438351

[B6] Boerboom W. Jacobs E. A. Khajeh L. van Kooten F. Ribbers G. M. Heijenbrok-Kal M. H. (2014). The relationship of coping style with depression, burden, and life dissatisfaction in caregivers of patients with subarachnoid haemorrhage. J. Rehabil. Med. 46, 321–326 10.2340/16501977-1273 24626873

[B7] Bouwknecht J. A. Paylor R. (2002). Behavioral and physiological mouse assays for anxiety: a survey in nine mouse strains. Behav. Brain Res. 136, 489–501 10.1016/S0166-4328(02)00200-0 12429412

[B8] Bowman S. Alvarez-Jimenez M. Wade D. McGorry P. Howie L. (2014). Forgotten family members: the importance of siblings in early psychosis. Early Interv. Psychiatry 8, 269–275 10.1111/eip.12068 23802612

[B9] Boyle M. P. Brewer J. A. Funatsu M. Wozniak D. F. Tsien J. Z. Izumi Y. (2005). Acquired deficit of forebrain glucocorticoid receptor produces depression-like changes in adrenal axis regulation and behavior. Proc. Natl. Acad. Sci. U.S.A. 102, 473–478 10.1073/pnas.0406458102 15623560 PMC544280

[B10] Branchi I. D'Andrea I. Cirulli F. Lipp H. P. Alleva E. (2010). Shaping brain development: mouse communal nesting blunts adult neuroendocrine and behavioral response to social stress and modifies chronic antidepressant treatment outcome. Psychoneuroendocrinology 35, 743–751 10.1016/j.psyneuen.2009.10.016 19945226

[B11] Branchi I. Santarelli S. Capoccia S. Poggini S. D'Andrea I. Cirulli F. (2013). Antidepressant treatment outcome depends on the quality of the living environment: a pre-clinical investigation in mice. PLoS ONE 8:e62226 10.1371/journal.pone.0062226 23653679 PMC3639948

[B12] Brown R. E. Mathieson W. B. Stapleton J. Neumann P. E. (1999). Maternal behavior in female C57BL/6J and DBA/2J inbred mice. Physiol. Behav. 67, 599–605 10.1016/S0031-9384(99)00109-2 10549899

[B13] Buwalda B. Kole M. H. Veenema A. H. Huininga M. De Boer S. F. Korte S. M. (2005). Long-term effects of social stress on brain and behavior: a focus on hippocampal functioning. Neurosci. Biobehav. Rev. 29, 83–97 10.1016/j.neubiorev.2004.05.005 15652257

[B14] Cabib S. Campus P. Colelli V. (2012). Learning to cope with stress: psychobiological mechanisms of stress resilience. Rev. Neurosci. 23, 659–672 10.1515/revneuro-2012-0080 23159866

[B15] Cabib S. Puglisi-Allegra S. Ventura R. (2002). The contribution of comparative studies in inbred strains of mice to the understanding of the hyperactive phenotype. Behav. Brain Res. 130, 103–109 10.1016/S0166-4328(01)00422-3 11864725

[B16] Campeau S. Liberzon I. Morilak D. Ressler K. (2011). Stress modulation of cognitive and affective processes. Stress 14, 503–519 10.3109/10253890.2011.596864 21790481 PMC3313908

[B17] Cantor-Graae E. (2007). The contribution of social factors to the development of schizophrenia: a review of recent findings. Can. J. Psychiatry 52, 277–286 10.3410/f.1116901.572995 17542378

[B18] Castren E. Voikar V. Rantamaki T. (2007). Role of neurotrophic factors in depression. Curr. Opin. Pharmacol. 7, 18–21 10.1016/j.coph.2006.08.009 17049922

[B19] Chourbaji S. Vogt M. A. Gass P. (2008). Mice that under- or overexpress glucocorticoid receptors as models for depression or posttraumatic stress disorder. Prog. Brain Res. 167, 65–77 10.1016/S0079-6123(07)67005-8 18037007

[B20] Crawley J. N. (2004). Designing mouse behavioral tasks relevant to autistic-like behaviors. Ment. Retard. Dev. Disabil. Res. Rev. 10, 248–258 10.1002/mrdd.20039 15666335

[B21] Crawley J. N. Belknap J. K. Collins A. Crabbe J. C. Frankel W. Henderson N. (1997). Behavioral phenotypes of inbred mouse strains: implications and recommendations for molecular studies. Psychopharmacology (Berl). 132, 107–124 10.1007/s002130050327 9266608

[B22] Curley J. P. Rock V. Moynihan A. M. Bateson P. Keverne E. B. Champagne F. A. (2010). Developmental shifts in the behavioral phenotypes of inbred mice: the role of postnatal and juvenile social experiences. Behav. Genet. 40, 220–232 10.1007/s10519-010-9334-4 20130977 PMC2862468

[B23] Del-Pino-Casado R. Frias-Osuna A. Palomino-Moral P. A. Pancorbo-Hidalgo P. L. (2011). Coping and subjective burden in caregivers of older relatives: a quantitative systematic review. J. Adv. Nurs. 67, 2311–2322 10.1111/j.1365-2648.2011.05725.x 21658096

[B24] Der-Avakian A. Markou A. (2012). The neurobiology of anhedonia and other reward-related deficits. Trends Neurosci. 35, 68–77 10.1016/j.tins.2011.11.005 22177980 PMC3253139

[B25] Devries A. C. (2002). Interaction among social environment, the hypothalamic-pituitary-adrenal axis, and behavior. Horm. Behav. 41, 405–413 10.1006/hbeh.2002.1780 12018936

[B26] Duff M. C. Gallegos D. R. Cohen N. J. Tranel D. (2013). Learning in Alzheimer's disease is facilitated by social interaction. J. Comp. Neurol. 521, 4356–4369 10.1002/cne.23433 23881834 PMC4038091

[B27] Ebstein R. P. Knafo A. Mankuta D. Chew S. H. Lai P. S. (2012). The contributions of oxytocin and vasopressin pathway genes to human behavior. Horm. Behav. 61, 359–379 10.1016/j.yhbeh.2011.12.014 22245314

[B28] Fish E. W. Riday T. T. McGuigan M. M. Faccidomo S. Hodge C. W. Malanga C. J. (2010). Alcohol, cocaine, and brain stimulation-reward in C57Bl6/J and DBA2/J mice. Alcohol. Clin. Exp. Res. 34, 81–89 10.1111/j.1530-0277.2009.01069.x 19860803 PMC5744672

[B29] Gamliel I. Yirmiya N. Jaffe D. H. Manor O. Sigman M. (2009). Developmental trajectories in siblings of children with autism: cognition and language from 4 months to 7 years. J. Autism. Dev. Disord. 39, 1131–1144 10.1007/s10803-009-0727-2 19326200 PMC2710494

[B30] Golden S. A. Covington H. E. 3rd. Berton O. Russo S. J. (2011). A standardized protocol for repeated social defeat stress in mice. Nat. Protoc. 6, 1183–1191 10.1038/nprot.2011.361 21799487 PMC3220278

[B32] Haller J. Baranyi J. Bakos N. Halasz J. (2004). Social instability in female rats: effects on anxiety and buspirone efficacy. Psychopharmacology (Berl) 174, 197–202 10.1007/s00213-003-1746-x 14760513

[B33] Hill M. N. Hellemans K. G. Verma P. Gorzalka B. B. Weinberg J. (2012). Neurobiology of chronic mild stress: parallels to major depression. Neurosci. Biobehav. Rev. 36, 2085–2117 10.1016/j.neubiorev.2012.07.001 22776763 PMC4821201

[B34] Holmes A. Wrenn C. C. Harris A. P. Thayer K. E. Crawley J. N. (2002). Behavioral profiles of inbred strains on novel olfactory, spatial and emotional tests for reference memory in mice. Genes Brain Behav. 1, 55–69 10.1046/j.1601-1848.2001.00005.x 12886950

[B35] Insel T. R. (2010). The challenge of translation in social neuroscience: a review of oxytocin, vasopressin, and affiliative behavior. Neuron 65, 768–779 10.1016/j.neuron.2010.03.005 20346754 PMC2847497

[B36] Jezova D. Skultetyova I. Tokarev D. I. Bakos P. Vigas M. (1995). Vasopressin and oxytocin in stress. Ann. N.Y. Acad. Sci. 771, 192–203 10.1111/j.1749-6632.1995.tb44681.x 8597399

[B37] Kaplan G. McCracken J. T. (2012). Psychopharmacology of autism spectrum disorders. Pediatr. Clin. North. Am. 59, 175–187 10.1016/j.pcl.2011.10.005 22284801

[B38] Karelina K. Devries A. C. (2011). Modeling social influences on human health. Psychosom. Med. 73, 67–74 10.1097/PSY.0b013e3182002116 21097660 PMC3076601

[B39] Karelina K. Norman G. J. Zhang N. Morris J. S. Peng H. Devries A. C. (2009). Social isolation alters neuroinflammatory response to stroke. Proc. Natl. Acad. Sci. U.S.A. 106, 5895–5900 10.1073/pnas.0810737106 19307557 PMC2667090

[B40] Karpova N. N. (2014). Role of BDNF epigenetics in activity-dependent neuronal plasticity. Neuropharmacology 76(Pt C), 709–718 10.1016/j.neuropharm.2013.04.002 23587647

[B41] Karpova N. N. Lindholm J. Pruunsild P. Timmusk T. Castren E. (2009). Long-lasting behavioural and molecular alterations induced by early postnatal fluoxetine exposure are restored by chronic fluoxetine treatment in adult mice. Eur. Neuropsychopharmacol. 19, 97–108 10.1016/j.euroneuro.2008.09.002 18973993

[B42] Kern R. S. Glynn S. M. Horan W. P. Marder S. R. (2009). Psychosocial treatments to promote functional recovery in schizophrenia. Schizophr. Bull. 35, 347–361 10.1093/schbul/sbn177 19176470 PMC2659313

[B43] Kessler R. C. Price R. H. Wortman C. B. (1985). Social factors in psychopathology: stress, social support, and coping processes. Annu. Rev. Psychol. 36, 531–572 10.1146/annurev.ps.36.020185.002531 3883893

[B44] Kiryk A. Mochol G. Filipkowski R. K. Wawrzyniak M. Lioudyno V. Knapska E. (2011). Cognitive abilities of Alzheimer's disease transgenic mice are modulated by social context and circadian rhythm. Curr. Alzheimer Res. 8, 883–892 10.2174/156720511798192745 22171952

[B45] Kobayashi Y. Sano Y. Vannoni E. Goto H. Suzuki H. Oba A. (2013). Genetic dissection of medial habenula-interpeduncular nucleus pathway function in mice. Front. Behav. Neurosci. 7:17 10.3389/fnbeh.2013.00017 23487260 PMC3594921

[B46] Krackow S. Vannoni E. Codita A. Mohammed A. H. Cirulli F. Branchi I. (2010). Consistent behavioral phenotype differences between inbred mouse strains in the IntelliCage. Genes Brain Behav. 9, 722–731 10.1111/j.1601-183X.2010.00606.x 20528956

[B47] Kulesskaya N. Rauvala H. Voikar V. (2011). Evaluation of social and physical enrichment in modulation of behavioural phenotype in C57BL/6J female mice. PLoS ONE 6:e24755 10.1371/journal.pone.0024755 21931844 PMC3169619

[B48] Kulesskaya N. Voikar V. Peltola M. Yegutkin G. G. Salmi M. Jalkanen S. (2013). CD73 is a major regulator of adenosinergic signalling in mouse brain. PLoS ONE 8:e66896 10.1371/journal.pone.0066896 23776700 PMC3680420

[B49] Kundakovic M. Lim S. Gudsnuk K. Champagne F. A. (2013). Sex-specific and strain-dependent effects of early life adversity on behavioral and epigenetic outcomes. Front. Psychiatry 4:78 10.3389/fpsyt.2013.00078 23914177 PMC3730082

[B50] Lipina T. V. Roder J. C. (2013). Co-learning facilitates memory in mice: a new avenue in social neuroscience. Neuropharmacology 64, 283–293 10.1016/j.neuropharm.2012.06.054 22776545

[B51] Lubin F. D. Roth T. L. Sweatt J. D. (2008). Epigenetic regulation of BDNF gene transcription in the consolidation of fear memory. J. Neurosci. 28, 10576–10586 10.1523/JNEUROSCI.1786-08.2008 18923034 PMC3312036

[B52] Lukas M. Neumann I. D. (2013). Oxytocin and vasopressin in rodent behaviors related to social dysfunctions in autism spectrum disorders. Behav. Brain Res. 251, 85–94 10.1016/j.bbr.2012.08.011 22981649

[B53] Magliano L. Fadden G. Economou M. Held T. Xavier M. Guarneri M. (2000). Family burden and coping strategies in schizophrenia: 1-year follow-up data from the BIOMED I study. Soc. Psychiatry Psychiatr. Epidemiol. 35, 109–115 10.1007/s001270050192 10855508

[B54] McFarlane H. G. Kusek G. K. Yang M. Phoenix J. L. Bolivar V. J. Crawley J. N. (2008). Autism-like behavioral phenotypes in BTBR T+tf/J mice. Genes Brain Behav. 7, 152–163 10.1111/j.1601-183X.2007.00330.x 17559418

[B55] McGowan P. O. Sasaki A. D'alessio A. C. Dymov S. Labonte B. Szyf M. (2009). Epigenetic regulation of the glucocorticoid receptor in human brain associates with childhood abuse. Nat. Neurosci. 12, 342–348 10.1038/nn.2270 19234457 PMC2944040

[B56] Moy S. S. Nadler J. J. Young N. B. Nonneman R. J. Segall S. K. Andrade G. M. (2008). Social approach and repetitive behavior in eleven inbred mouse strains. Behav. Brain Res. 191, 118–129 10.1016/j.bbr.2008.03.015 18440079 PMC2441761

[B57] Moy S. S. Nadler J. J. Young N. B. Perez A. Holloway L. P. Barbaro R. P. (2007). Mouse behavioral tasks relevant to autism: phenotypes of 10 inbred strains. Behav. Brain Res. 176, 4–20 10.1016/j.bbr.2006.07.030 16971002 PMC1857288

[B58] Mozhui K. Karlsson R. M. Kash T. L. Ihne J. Norcross M. Patel S. (2010). Strain differences in stress responsivity are associated with divergent amygdala gene expression and glutamate-mediated neuronal excitability. J. Neurosci. 30, 5357–5367 10.1523/JNEUROSCI.5017-09.2010 20392957 PMC2866495

[B59] Naert G. Ixart G. Maurice T. Tapia-Arancibia L. Givalois L. (2011). Brain-derived neurotrophic factor and hypothalamic-pituitary-adrenal axis adaptation processes in a depressive-like state induced by chronic restraint stress. Mol. Cell. Neurosci. 46, 55–66 10.1016/j.mcn.2010.08.006 20708081

[B60] Nestler E. J. Barrot M. Dileone R. J. Eisch A. J. Gold S. J. Monteggia L. M. (2002). Neurobiology of depression. Neuron 34, 13–25 10.1016/S0896-6273(02)00653-0 11931738

[B61] O'Brien I. Duffy A. Nicholl H. (2009). Impact of childhood chronic illnesses on siblings: a literature review. Br. J. Nurs. 18, 1358, 1360–1365 10.12968/bjon.2009.18.22.45562 20081690

[B62] Olivier B. Leahy C. Mullen T. Paylor R. Groppi V. E. Sarnyai Z. (2001). The DBA/2J strain and prepulse inhibition of startle: a model system to test antipsychotics? Psychopharmacology (Berl). 156, 284–290 10.1007/s002130100828 11549230

[B64] Papaleo F. Silverman J. L. Aney J. Tian Q. Barkan C. L. Chadman K. K. (2011). Working memory deficits, increased anxiety-like traits, and seizure susceptibility in BDNF overexpressing mice. Learn. Mem. 18, 534–544 10.1101/lm.2213711 21791566 PMC3256571

[B65] Pattij T. Janssen M. C. Loos M. Smit A. B. Schoffelmeer A. N. Van Gaalen M. M. (2007). Strain specificity and cholinergic modulation of visuospatial attention in three inbred mouse strains. Genes Brain Behav. 6, 579–587 10.1111/j.1601-183X.2006.00284.x 17116168

[B66] Perkovic N. M. Erjavec N. G. Zivkovic M. Sagud M. Uzun S. Mihaljevic-Peles A. (2014). Association between the brain-derived neurotrophic factor Val66Met polymorphism and therapeutic response to olanzapine in schizophrenia patients. Psychopharmacology (Berl). [Epub ahead of print]. 10.1007/s00213-014-3515-4 24595507

[B67] Pinkston J. W. Lamb R. J. (2011). Delay discounting in C57BL/6J and DBA/2J mice: adolescent-limited and life-persistent patterns of impulsivity. Behav. Neurosci. 125, 194–201 10.1037/a0022919 21463022 PMC3198828

[B68] Pulkki-Raback L. Kivimaki M. Ahola K. Joutsenniemi K. Elovainio M. Rossi H. (2012). Living alone and antidepressant medication use: a prospective study in a working-age population. BMC Public Health 12:236 10.1186/1471-2458-12-236 22443226 PMC3338384

[B69] Razzoli M. Carboni L. Andreoli M. Ballottari A. Arban R. (2011). Different susceptibility to social defeat stress of BalbC and C57BL6/J mice. Behav. Brain Res. 216, 100–108 10.1016/j.bbr.2010.07.014 20654656

[B70] Reichow B. Volkmar F. R. (2010). Social skills interventions for individuals with autism: evaluation for evidence-based practices within a best evidence synthesis framework. J. Autism Dev. Disord. 40, 149–166 10.1007/s10803-009-0842-0 19655240

[B71] Sadowsky C. H. Galvin J. E. (2012). Guidelines for the management of cognitive and behavioral problems in dementia. J. Am. Board Fam. Med. 25, 350–366 10.3122/jabfm.2012.03.100183 22570399

[B72] Sapolsky R. M. Meaney M. J. McEwen B. S. (1985). The development of the glucocorticoid receptor system in the rat limbic brain. III. Negative-feedback regulation. Brain Res. 350, 169–173 10.1016/0165-3806(85)90261-5 3986611

[B73] Satoh Y. Endo S. Nakata T. Kobayashi Y. Yamada K. Ikeda T. (2011). ERK2 contributes to the control of social behaviors in mice. J. Neurosci. 31, 11953–11967 10.1523/JNEUROSCI.2349-11.2011 21849556 PMC6623182

[B74] Schmidt M. V. Scharf S. H. Liebl C. Harbich D. Mayer B. Holsboer F. (2010). A novel chronic social stress paradigm in female mice. Horm. Behav. 57, 415–420 10.1016/j.yhbeh.2010.01.010 20100488

[B75] Schmidt M. V. Sterlemann V. Ganea K. Liebl C. Alam S. Harbich D. (2007). Persistent neuroendocrine and behavioral effects of a novel, etiologically relevant mouse paradigm for chronic social stress during adolescence. Psychoneuroendocrinology 32, 417–429 10.1016/j.psyneuen.2007.02.011 17449187

[B76] Shaked M. Gamliel I. Yirmiya N. (2006). Theory of mind abilities in young siblings of children with autism. Autism 10, 173–187 10.1177/1362361306062023 16613866

[B77] Shanks N. Anisman H. (1988). Stressor-provoked behavioral changes in six strains of mice. Behav. Neurosci. 102, 894–905 10.1037/0735-7044.102.6.894 3214540

[B78] Sharpe D. Rossiter L. (2002). Siblings of children with a chronic illness: a meta-analysis. J. Pediatr. Psychol. 27, 699–710 10.1093/jpepsy/27.8.699 12403860

[B79] Singer P. Feldon J. Yee B. K. (2009). Are DBA/2 mice associated with schizophrenia-like endophenotypes? A behavioural contrast with C57BL/6 mice. Psychopharmacology (Berl) 206, 677–698 10.1007/s00213-009-1568-6 19484222

[B80] Slattery D. A. Uschold N. Magoni M. Bar J. Popoli M. Neumann I. D. (2012). Behavioural consequences of two chronic psychosocial stress paradigms: anxiety without depression. Psychoneuroendocrinology 37, 702–714 10.1016/j.psyneuen.2011.09.002 21962377

[B81] Sokolowska E. Kängsep S. Misiewicz Z. Voikar V. Hovatta I. (2013). Behavioral Outcome of Chronic Social Defeat in Four Inbred Mouse Strains. Program No. 543.17.2013 Neuroscience Meeting Planner. San Diego, CA: Society for Neuroscience (Online).

[B82] Soliman F. Glatt C. E. Bath K. G. Levita L. Jones R. M. Pattwell S. S. (2010). A genetic variant BDNF polymorphism alters extinction learning in both mouse and human. Science 327, 863–866 10.1126/science.1181886 20075215 PMC2829261

[B83] Southwick S. M. Vythilingam M. Charney D. S. (2005). The psychobiology of depression and resilience to stress: implications for prevention and treatment. Annu. Rev. Clin. Psychol. 1, 255–291 10.1146/annurev.clinpsy.1.102803.143948 17716089

[B84] Steele A. Maruyama N. Galynker I. (2010). Psychiatric symptoms in caregivers of patients with bipolar disorder: a review. J. Affect. Disord. 121, 10–21 10.1016/j.jad.2009.04.020 19443040

[B85] Tolliver B. K. Carney J. M. (1994). Sensitization to stereotypy in DBA/2J but not C57BL/6J mice with repeated cocaine. Pharmacol. Biochem. Behav. 48, 169–173 10.1016/0091-3057(94)90513-4 8029287

[B86] Tulogdi A. Toth M. Barsvari B. Biro L. Mikics E. Haller J. (2014). Effects of resocialization on post-weaning social isolation-induced abnormal aggression and social deficits in rats. Dev. Psychobiol. 56, 49–57 10.1002/dev.21090 23168609

[B87] Upchurch M. Wehner J. M. (1988). DBA/2Ibg mice are incapable of cholinergically-based learning in the Morris water task. Pharmacol. Biochem. Behav. 29, 325–329 10.1016/0091-3057(88)90164-5 3362927

[B88] Van Os J. Mcguffin P. (2003). Can the social environment cause schizophrenia? Br. J. Psychiatry 182, 291–292 10.1192/bjp.182.4.291 12668402

[B89] van Tilborg I. A. Kessels R. P. Hulstijn W. (2011). Learning by observation and guidance in patients with Alzheimer's dementia. NeuroRehabilitation 29, 295–304 10.3233/NRE-2011-0705 22142763

[B90] Voikar V. Polus A. Vasar E. Rauvala H. (2005). Long-term individual housing in C57BL/6J and DBA/2 mice: assessment of behavioral consequences. Genes Brain Behav. 4, 240–252 10.1111/j.1601-183X.2004.00106.x 15924556

[B91] Walker M. Fureix C. Palme R. Mason G. (2013). Co-housing rodents with different coat colours as a simple, non-invasive means of individual identification: validating mixed-strain housing for C57BL/6 and DBA/2 mice. PLoS ONE 8:e77541 10.1371/journal.pone.0077541 24204864 PMC3810273

[B92] Wang J. Fanous S. Terwilliger E. F. Bass C. E. Hammer R. P. Jr. Nikulina E. M. (2013). BDNF overexpression in the ventral tegmental area prolongs social defeat stress-induced cross-sensitization to amphetamine and increases DeltaFosB expression in mesocorticolimbic regions of rats. Neuropsychopharmacology 38, 2286–2296 10.1038/npp.2013.130 23689674 PMC3773680

[B93] Weinberger S. B. Koob G. F. Martinez J. L. Jr. (1992). Differences in one-way active avoidance learning in mice of three inbred strains. Behav. Genet. 22, 177–188 10.1007/BF01066996 1596257

[B94] Wittenberg E. Ritter G. A. Prosser L. A. (2013). Evidence of spillover of illness among household members: EQ-5D scores from a US sample. Med. Decis. Making 33, 435–443 10.1177/0272989X12464434 23100461 PMC3606811

[B95] Yang M. Perry K. Weber M. D. Katz A. M. Crawley J. N. (2011). Social peers rescue autism-relevant sociability deficits in adolescent mice. Autism Res. 4, 17–27 10.1002/aur.163 20928844 PMC3065860

[B98] Yang M. Zhodzishsky V. Crawley J. N. (2007). Social deficits in BTBR T+tf/J mice are unchanged by cross-fostering with C57BL/6J mothers. Int. J. Dev. Neurosci. 25, 515–521 10.1016/j.ijdevneu.2007.09.008 17980995 PMC2194756

[B96] Zhang L. Benedek D. M. Fullerton C. S. Forsten R. D. Naifeh J. A. Li X. X. (2013a). PTSD risk is associated with BDNF Val66Met and BDNF overexpression. Mol. Psychiatry 19, 8–10 10.1038/mp.2012.180 23319005

[B97] Zhang T. Y. Labonte B. Wen X. L. Turecki G. Meaney M. J. (2013b). Epigenetic mechanisms for the early environmental regulation of hippocampal glucocorticoid receptor gene expression in rodents and humans. Neuropsychopharmacology 38, 111–123 10.1038/npp.2012.149 22968814 PMC3521971

